# Phase Relations in the *Ln*_2_O_3_–Cr_2_O_3_–B_2_O_3_ (*Ln* = Gd–Lu) Ternary Oxide Systems

**DOI:** 10.3390/ma16051831

**Published:** 2023-02-23

**Authors:** Nikolai Kuzmin, Victor Maltsev, Elizaveta Mikliaeva, Elena Volkova, Kirill Boldyrev, Elizaveta Koporulina

**Affiliations:** 1 Faculty of Geology, Moscow State University, 119991 Moscow, Russia; 2 Institute of Spectroscopy, Russian Academy of Science, Troitsk, 108840 Moscow, Russia; 3 Landau Phystech School of Physics and Research, Moscow Institute of Physics and Technology, 141701 Dolgoprudny, Russia; 4 Branch “Aprelevka Department of VNIGNI”, All-Russian Research Geological Oil Institute, 143360 Aprelevka, Russia; 5 Melnikov Research Institute of Comprehensive Exploitation of Mineral Resources, Russian Academy of Science, 111020 Moscow, Russia

**Keywords:** phase relations, rare-earth chromium borates, solid-state reaction, powder X-ray diffraction, differential scanning calorimetry

## Abstract

In this work, isothermal sections of the *Ln*_2_O_3_–Cr_2_O_3_–B_2_O_3_ (*Ln* = Gd–Lu) ternary oxide systems at 900, 1000, and 1100 °C were constructed by determining the phase relations by using a powder X-ray diffraction technique. As a result, these systems were divided into subsidiary subsystems. Two types of double borates, *Ln*Cr_3_(BO_3_)_4_ (*Ln* = Gd–Er) and *Ln*Cr(BO_3_)_2_ (*Ln* = Ho–Lu), were observed in the investigated systems. Regions of phase stability for *Ln*Cr_3_(BO_3_)_4_ and *Ln*Cr(BO_3_)_2_ were determined. It was shown that the *Ln*Cr_3_(BO_3_)_4_ compounds crystallized in rhombohedral and monoclinic polytype modifications up to 1100 °C; above this temperature and up to the melting points, the monoclinic modification was predominantly formed. The *Ln*Cr_3_(BO_3_)_4_ (*Ln* = Gd–Er) and *Ln*Cr(BO_3_)_2_ (*Ln* = Ho–Lu) compounds were characterized by using a powder X-ray diffraction method and thermal analysis.

## 1. Introduction

The *Ln*_2_O_3_–Cr_2_O_3_–B_2_O_3_ (*Ln* = Gd–Lu) ternary oxide systems have not been studied so far in terms of their phase relations in an air atmosphere. However, compounds synthesized in binary systems corresponding to the sides of these ternary diagrams are of great interest in terms of both their crystal structures and their physicochemical properties.

The reactivity of the binary oxide systems of *Ln*_2_O_3_–B_2_O_3_, Cr_2_O_3_–B_2_O_3_, and *Ln*_2_O_3_–Cr_2_O_3_ has been analyzed in a number of publications, although some of these systems have not yet been sufficiently investigated. Most studies refer to binary diagrams of *Ln*_2_O_3_–B_2_O_3_ (*Ln* = Y, La–Nd, Sm–Lu), where the compounds of *Ln*(BO_2_)_3_, *Ln*BO_3_, *Ln*_3_BO_6_, and *Ln*B_5_O_9_ are described [1,2,3,4]. Lanthanide metaborates, *Ln*(BO_2_)_3_ (*Ln* = La–Nd, Sm–Dy), exhibit two structural modifications under normal pressure: α-*Ln*(BO_2_)_3_, where *Ln* = La–Nd, Sm–Tb (space group (sp. gr.) *I*2/*a*) [5,6,7,8,9,10,11], and *β*-*Ln*(BO_2_)_3_, where *Ln* = Tb–Dy with sp. gr. *Pnma* [12,13]. It was found that the metaborates of *Ln*(BO_2_)_3_ (*Ln* = La, Nd) melt congruently, while the metaborates of *Ln*(BO_2_)_3_ (*Ln* = Sm–Gd) melt incongruently at 1000–1150 °C [1,2]. In the latter case, melting occurs with the formation of *Ln*BO_3_ (*Ln* = Eu, Gd) and B_2_O_3_ [1]. Rare-earth orthoborates, *Ln*BO_3_ (*Ln* = Y, La–Nd, Sm–Lu), are isostructural to three CaCO_3_ modifications at normal pressure depending on the *Ln*-cation: aragonite (*λ*-*Ln*BO_3_, with *Ln* = La–Nd), vaterite (*π*- and/or *μ*-*Ln*BO_3_, with *Ln* = Y, Sm–Lu), and calcite (*β*-LuBO_3_) [1,14,15,16,17]. They melt congruently in the temperature range of 1600–1700 °C [1,2]. Rare-earth oxyborates with the general formula of *Ln*_3_BO_6_ (*Ln* = Y, La–Nd, Sm–Lu) are the most understudied compounds in this diagram. Initially, oxyborates were found in the La_2_O_3_–B_2_O_3_ system, and their formula was determined as *Ln*_3_BO_6_ based on phase identification [2]. However, the structural formulas of *Ln*_3_BO_6_ (*Ln* = La, Nd), *Ln*_3_BO_6_ (*Ln* = Y, Gd), and Yb_3_BO_6_ were further revised as *Ln*_26_(BO_3_)_8_O_27_ (*Ln* = La, Nd) [18,19], *Ln*_17.33_(BO_3_)_4_(B_2_O_5_)_2_O_16_ (*Ln* = Y, Gd) [20,21], and Yb_26_(BO_3_)_4_(B_2_O_5_)_2_(B_4_O_11_)O_24_ [22]. Thus, the oxyborate family is divided into three groups: *Ln*_3_BO_6_ (*Ln* = La–Nd) with sp. gr. *P*2_1_/*c*, *Ln*_3_BO_6_ (*Ln* = Y, Sm–Tm) with sp. gr. *Cm*, and *Ln*_3_BO_6_ (*Ln* = Yb, Lu) with sp. gr. *C*2/*m*. Levin et al. [2] found that La_3_BO_6_ oxyborate melts incongruently at 1386 °C. Lanthanide pentaborates, *Ln*B_5_O_9_ (*Ln* = La–Nd, Sm–Er), were obtained through the decomposition of *Ln*[B_8_O_11_(OH)_5_] (*Ln* = La–Nd), *Ln*[B_9_O_13_(OH)_4_] (*Ln* = Pr, Nd, Sm, Eu), and *Ln*[B_6_O_9_(OH)_3_] (*Ln* = Sm–Er) [3,4]. These compounds have two structural modifications: *β*-*Ln*B_5_O_9_ (*Ln* = La, Ce) (sp. gr. *P*2_1_/*c*) and *α*-*Ln*B_5_O_9_ (*Ln* = Pr, Nd, Sm–Er) (sp. gr. *I*4_1_/*acd*). Pentaborates decompose at 800–900 °C into metaborates *Ln*(BO_2_)_3_ (*Ln* = Sm–Tb) and orthoborates *Ln*BO_3_ (*Ln* = Dy–Er). The representatives of the *Ln*_2_O_3_–B_2_O_3_ system are considered to be promising candidates for luminescent and nonlinear optical materials [23,24,25].

The binary system of Cr_2_O_3_–B_2_O_3_ has been investigated less. Two solid phases, CrBO_3_ [26] and Cr_3_BO_6_ [27], were reported to exist in it. Chromium orthoborate, CrBO_3_, has a calcite-type structure (sp. gr. $R\bar{3}c$) and decomposes at 1220 °C to form Cr_2_O_3_ and B_2_O_3_. Bither et al. [28] reported that CrBO_3_ appeared to be a low-temperature antiferromagnetic material with the Néel temperature (*T_N_*) of 15 K. Chromium oxyborate, Cr_3_BO_6_, crystallizes in the sp. gr. *Pnma*. This compound demonstrates low thermal stability in air and decomposes into Cr_2_O_3_ and CrBO_3_ at 800 °C. The electrochemical properties of these materials were investigated, and it was shown that Cr_3_BO_6_ could be interesting as a negative electrode for lithium-ion batteries [27].

According to data from the literature, only *Ln*CrO_3_ phases were found in the binary systems of *Ln*_2_O_3_–Cr_2_O_3_ (*Ln* = Y, La–Nd, Sm–Lu) [29]. They exhibited perovskite-type structure with the sp. gr. *Pbnm* [30,31] and demonstrated high congruent melting points at 2300–2400 °C [32]. These compounds are antiferromagnetically ordered with *T_N_* in the temperature range of 110–290 K [33,34]. Multiferroic properties were observed for some rare-earth orthochromites [35,36]. *Ln*CrO_3_ materials are *p*-type semiconductors, which are valuable for sensor applications [37].

In the *Ln*_2_O_3_–Cr_2_O_3_–B_2_O_3_ (*Ln* = Y, La–Nd, Sm–Lu) ternary systems, two double borates were found: *Ln*Cr_3_(BO_3_)_4_ with a huntite-type structure (sp. gr. *R*32) [38,39] and *Ln*Cr(BO_3_)_2_ with a dolomite-type structure (sp. gr. $R\bar{3}$) [40,41]. Both borate families are still poorly studied. Rare-earth chromium borates of the huntite family were synthesized from multicomponent flux melts [42] and through solid-state reactions [43]. In addition to their rhombohedral structure, *Ln*Cr_3_(BO_3_)_4_ compounds also have a monoclinic modification with sp. gr. *C*2/*c* [44,45,46]. These materials have been found to exhibit antiferromagnetic ordering with *T_N_* in the temperature range of 6.5–10 K [43,47,48,49]. Several studies of *Ln*Cr(BO_3_)_2_ compounds have been performed, including the refinement of their crystal structure and measurements of antiferromagnetic ordering temperatures (6.1–8.1 K) [50,51].

The great variety of properties specific to borates appearing both in binary *Ln*_2_O_3_–B_2_O_3_ (*Ln* = Gd–Lu), Cr_2_O_3_–B_2_O_3_, and *Ln*_2_O_3_–Cr_2_O_3_ (*Ln* = Gd–Lu) and in ternary *Ln*_2_O_3_–Cr_2_O_3_–B_2_O_3_ (*Ln* = Gd–Lu) diagrams makes such systems interesting and challenging for further investigation. Therefore, the purpose of this work is to study the phase relations in *Ln*_2_O_3_–Cr_2_O_3_–B_2_O_3_ (*Ln* = Gd–Lu) ternary systems, the phase formation of promising magnetic materials in them, and the determination of the structural and thermal properties of the latter.

## 2. Materials and Methods

The ternary diagrams were studied through the solid-state reaction (SSR) in a high-temperature muffle furnace in air. The initial reagents were oxides (*Ln*_2_O_3_ (*Ln* = Gd, Dy–Lu) (99.9%), Tb_4_O_7_ (99.9%), Cr_2_O_3_ (99.9%)) and orthoboric acid (H_3_BO_3_ (99.9%)). The oxides were preliminarily calcined before mixing to remove water. After that, they were mixed with orthoboric acid in the required proportions in an agate mortar. A 5 mol.% excess of H_3_BO_3_ was added to compensate for the loss of B_2_O_3_ due to volatilization during heating. This mixture was pressed into tablets (12 mm in diameter) and held in alumina crucibles at 400 °C to decompose the orthoboric acid. Then, the mixture was ground again in acetone, heated at a rate of 4 °C/min, and held at 24 h in the temperature range of 800–1300 °C. After each heating stage, the samples were left in the furnace until they cooled to room temperature; then, the phase composition was determined with a powder X-ray diffraction (PXRD) method. The heating was repeated until the PXRD patterns remained absolutely stable. New tablets were used for each sintering temperature.

PXRD was carried out by using an ARL X’tra diffractometer (Thermo Fisher Scientific, Basel, Switzerland) equipped with a MYTHEN2 R 1D detector (Dectris, Baden-Daettwil, Switzerland). The diffraction patterns of the SSR products were recorded in continuous mode at room temperature by using CuK_α1,2_ radiation (λ = 1.540562 Å) in the range of 10° ≤ 2θ ≤ 70° with a scan speed of 8° per minute, U = 40 kV, and I = 40 mA. Long PXRD scans were collected in the range of 10° ≤ 2θ ≤ 90° for *Ln*Cr_3_(BO_3_)_4_ and *Ln*Cr(BO_3_)_2_ to carry out a quantitative analysis.

Compounds were identified by matching the experimental PXRD patterns to those of the ICDD PDF-2 powder diffraction database release 2020 [52].

The refinement of the lattice parameters of the rare-earth chromium borates and dysprosium metaborate was carried out through the Le Bail method by using the JANA2006 software package [53]. All parameters were refined with the least-square method. The pseudo-Voigt function was used as the peak profile function. The initial structural parameters were the structural data for DyCr_3_(BO_3_)_4_ (monoclinic, sp. gr. *C*2/*c*, *a* = 7.394 Å, *b* = 9.450 Å, *c* = 11.357 Å, *α* = *γ* = 90°, *β* = 103.9°; rhombohedral, sp. gr. *R*32, *a* = 9.461 Å, *c* = 7.488 Å, *α* = *β* = 90°; *γ* = 120°) [49] and *α*-Tb(BO_2_)_3_ (monoclinic, sp. gr. *C*2/*c*, *a* = 6.21781(5) Å, *b* = 8.02564(6) Å, *c* = 7.80659(4) Å, *α* = *γ* = 90°, *β* = 93.6935(7)°) [11].

Differential scanning calorimetry (DSC) was performed by means of STA 449 F5 Jupiter equipment (Netzsch, Selb, Germany) in the temperature range of 50–1500 °C with a heating rate of 20 °C/min in Ar gas flow. PtRh crucibles were used in the DSC experiments.

## 3. Results and Discussion

### 3.1. Phase Formation in the Ln_2_O_3_–Cr_2_O_3_–B_2_O_3_ (Ln = Gd–Lu) Systems

#### 3.1.1. *Ln*_2_O_3_–Cr_2_O_3_–B_2_O_3_ (*Ln* = Gd, Tb) Ternary Systems

**Figure 1:**
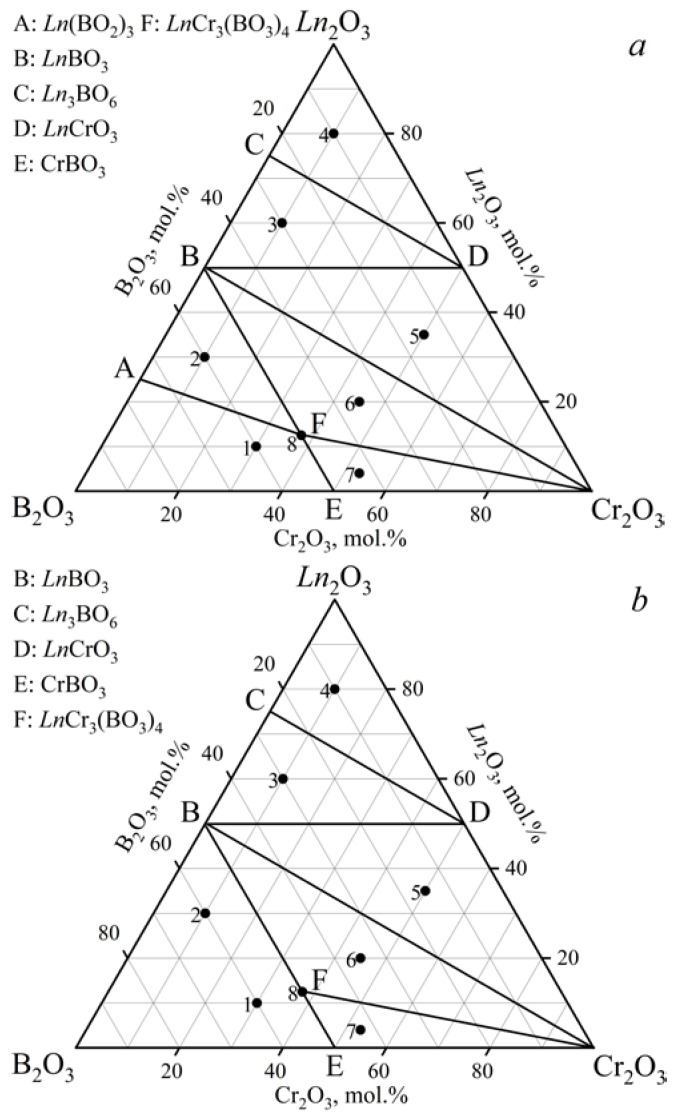
Phase relations in the systems: (**a**) Gd_2_O_3_–Cr_2_O_3_–B_2_O_3_ at 900 and 1000 °C, Tb_2_O_3_–Cr_2_O_3_–B_2_O_3_ at 900 °C; (**b**) Gd_2_O_3_–Cr_2_O_3_–B_2_O_3_ at 1100 °C, Tb_2_O_3_–Cr_2_O_3_–B_2_O_3_ at 1000 and 1100 °C.

The phase relations in the *Ln*_2_O_3_–Cr_2_O_3_–B_2_O_3_ (*Ln* = Gd, Tb) ternary systems at 900, 1000, and 1100 °C (Figure 1, Table 1) were determined based on an analysis of 24 samples (eight samples per isothermal section). Each diagram had seven different subsidiary subsystems. Double borates *Ln*Cr_3_(BO_3_)_4_ (GdCr_3_(BO_3_)_4_ [PDF-2 #01-085-4144]) were established in both systems. Metaborates (Gd(BO_2_)_3_ [PDF-2 #01-086-3642] and Tb(BO_2_)_3_ [PDF-2 #01-072-3639]) crystallized in the *α*-modification. There was no *α*-Gd(BO_2_)_3_ compound in the cross-section at 1100 °C (Figure 1b), which was due to its melting at 1085 °C [54]. The metaborate *α*-Tb(BO_2_)_3_ was not observed at temperatures above 900 °C due to its lower melting point than that of *α*-Gd(BO_2_)_3_. The results obtained correlated well with the decrease in the melting point in the *Ln*(BO_2_)_3_ series from La to Gd [1,2]. Orthoborates (GdBO_3_ [PDF-2 #01-089-6546] and TbBO_3_ [PDF-2 #01-080-3937]) crystallized in a vaterite modification. The data from the PDF-2 #01-074-3085 card were used to determine the Gd_2_O_3_ phase. Oxyborates *Ln*_3_BO_6_ (*Ln* = Gd, Tb) were identified based on the PXRD data of the isostructural compound Y_3_BO_6_ [PDF-2 #00-034-0291].

**Figure 2:**
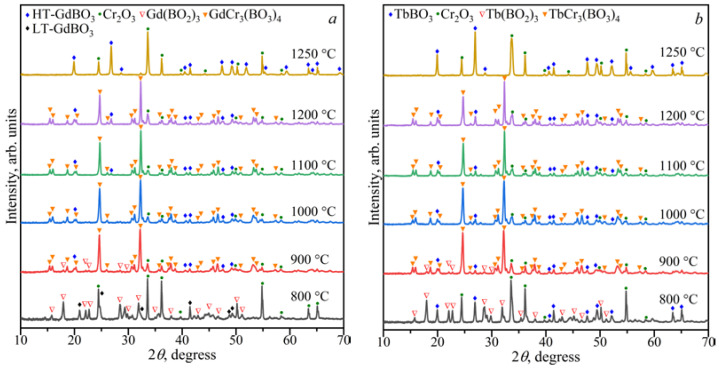
PXRD patterns of the compositions of Gd_2_O_3_:3Cr_2_O_3_:4B_2_O_3_ (**a**) and Tb_2_O_3_:3Cr_2_O_3_:4B_2_O_3_ (**b**) in the temperature range of 800–1250 °C.

The stability of the *Ln*Cr_3_(BO_3_)_4_ compounds in the range of 800–1250 °C was investigated (Figure 2). Double borates of *Ln*Cr_3_(BO_3_)_4_ coexisted with *Ln*BO_3_, *α*-*Ln*(BO_2_)_3_, and Cr_2_O_3_ at 900 °C, and in the temperature range of 1000–1200 °C, they existed with *Ln*BO_3_ and Cr_2_O_3_. The compounds of *Ln*BO_3_ and Cr_2_O_3_ [PDF-2 #00-038-1479] were observed at temperatures of 1250 °C, while *Ln*BO_3_, *α*-*Ln*(BO_2_)_3_, and Cr_2_O_3_ were formed at 800 °C.

Thus, in the investigated ternary systems, the reactions proceeded according to the following equations:

*Ln*_2_O_3_ + B_2_O_3_ → 2*Ln*BO_3_ (800–1250 °C) (1)

*Ln*_2_O_3_ + 3B_2_O_3_ → 2α-*Ln*(BO_2_)_3_ (800–1000 °C for *Ln* = Gd, 800–900 °C for *Ln* = Tb) (2)

*Ln*_2_O_3_ + 3Cr_2_O_3_ + 4B_2_O_3_ → 2*Ln*Cr_3_(BO_3_)_4_ (900–1200 °C) (3)

Figure 2a shows that the borate GdBO_3_ crystallized in the low-temperature (LT, PDF-2 #01-089-6545) modification at 800 °C, while the high-temperature (HT) vaterite modification of GdBO_3_ formed above 900 °C. This was consistent with the data of Ren et al. [17].

#### 3.1.2. The Dy_2_O_3_–Cr_2_O_3_–B_2_O_3_ Ternary System

**Figure 3:**
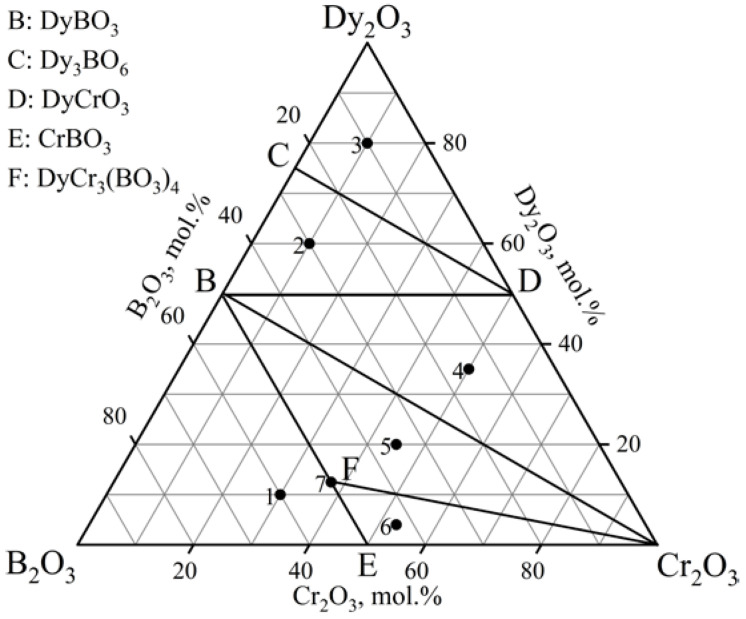
Phase relations in the Dy_2_O_3_–Cr_2_O_3_–B_2_O_3_ system at 900, 1000, and 1100 °C.

The phase relations in the Dy_2_O_3_–Cr_2_O_3_–B_2_O_3_ ternary system at 900, 1000, and 1100 °C (Figure 3, Table 2) were determined based on 21 experimental compositions (seven specimens for each isothermal section). The system exhibited six subsidiary subsystems. The dysprosium chromium borate, DyCr_3_(BO_3_)_4_, was observed. DyBO_3_ [PDF-2 #01-074-1933] demonstrated the vaterite modification. The powder data from the PDF-2 #01-083-9837 card were used to determine the Dy_2_O_3_ phase. Dy(BO_2_)_3_ was determined from the similarity with the PXRD patterns of known metaborates of *Ln*(BO_2_)_3_ (*Ln* = Gd, Tb). The oxyborate Dy_3_BO_6_ was isostructural with Y_3_BO_6_. All compounds established in the considered diagram were stable up to 1100 °C.

Vicat et al. [40] reported the synthesis of the double borate DyCr(BO_3_)_2_ with a dolomite-type structure. However, the existence of this phase is doubtful. For example, Doi et al. [50] synthesized only *Ln*Cr(BO_3_)_2_ (*Ln* = Y, Ho—Lu) borates. Our results confirm these data.

**Figure 4:**
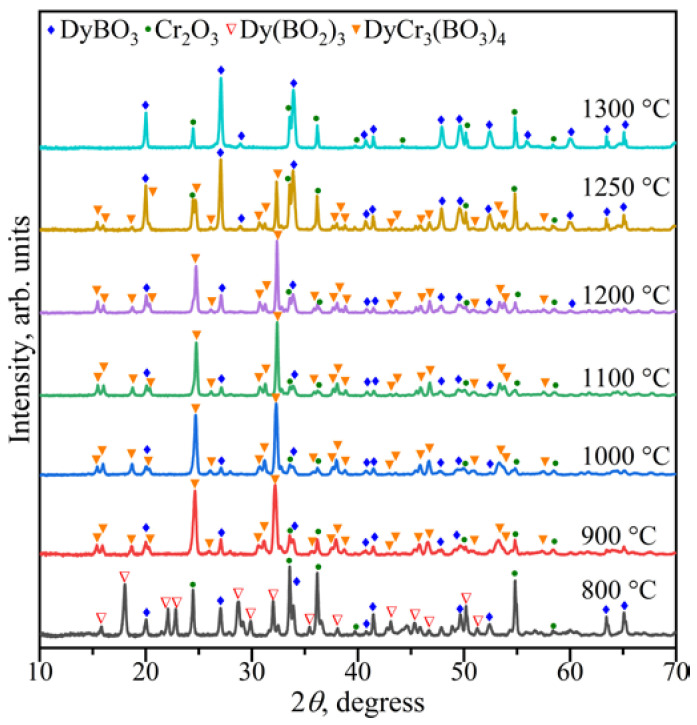
PXRD patterns of the Dy_2_O_3_: 3Cr_2_O_3_:4B_2_O_3_ composition in the temperature range of 800–1300 °C.

The study of the stability of DyCr_3_(BO_3_)_4_ was carried out in the range of 800–1300 °C. It was found that above 1250 °C, DyBO_3_ and Cr_2_O_3_ were formed (Figure 4). DyCr_3_(BO_3_)_4_ coexisted with DyBO_3_ and Cr_2_O_3_ in the range of 900–1250 °C. The compounds DyBO_3_, *α*-Dy(BO_2_)_3_, and Cr_2_O_3_ were observed below 900 °C.

Consequently, the following reactions proceeded:

Dy_2_O_3_ + B_2_O_3_ → 2DyBO_3_ (800–1300 °C) (4)

Dy_2_O_3_ + 3B_2_O_3_ → 2*α*-Dy(BO_2_)_3_ (800 °C) (5)

Dy_2_O_3_ + 3Cr_2_O_3_ + 4B_2_O_3_ → 2DyCr_3_(BO_3_)_4_ (900–1250 °C) (6)

#### 3.1.3. The *Ln*_2_O_3_–Cr_2_O_3_–B_2_O_3_ (*Ln* = Ho, Er) Ternary Systems

**Figure 5:**
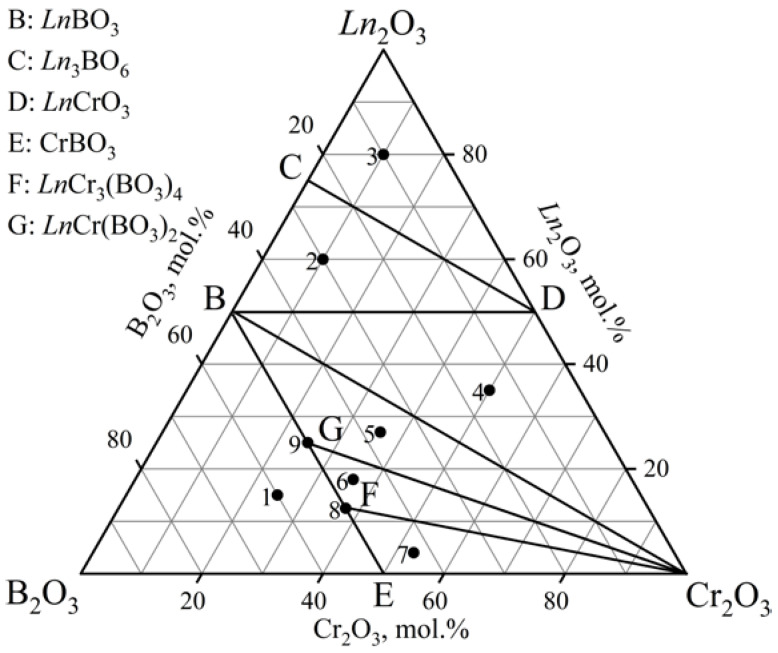
Phase relations in the *Ln*_2_O_3_–Cr_2_O_3_–B_2_O_3_ (*Ln* = Ho, Er) system at 900, 1000, and 1100 °C.

The phase relations in the *Ln*_2_O_3_–Cr_2_O_3_–B_2_O_3_ (*Ln* = Ho, Er) ternary systems at 900, 1000, and 1100 °C (Figure 5, Table 3) on the basis of 27 samples (nine specimens per isothermal section) were investigated. There were seven subsidiary subsystems in each of the systems considered. *Ln*Cr_3_(BO_3_)_4_ and *Ln*Cr(BO_3_)_2_ (HoCr(BO_3_)_2_ [PDF-2 #01-082-9333] and ErCr(BO_3_)_2_ [PDF-2 #01-082-9334]) borates were found. The orthoborates (HoBO_3_ [PDF-2 #01-074-1934] and ErBO_3_ [PDF-2 #01-013-0486] were isostructural to the vaterite modification. The Ho_2_O_3_ and Er_2_O_3_ phases were determined by using the powder data from the PDF-2 #01-074-3085 and PDF-2 #00-013-0387 cards, respectively. The PXRD data of the isostructural compound Y_3_BO_6_ were used to determine the *Ln*_3_BO_6_ phases. All compounds of these systems were stable up to 1100 °C.

Our results confirm the existence of the ErCr_3_(BO_3_)_4_ compound, which was reported only by Kurazhkovskaya et al. [45].

**Figure 6:**
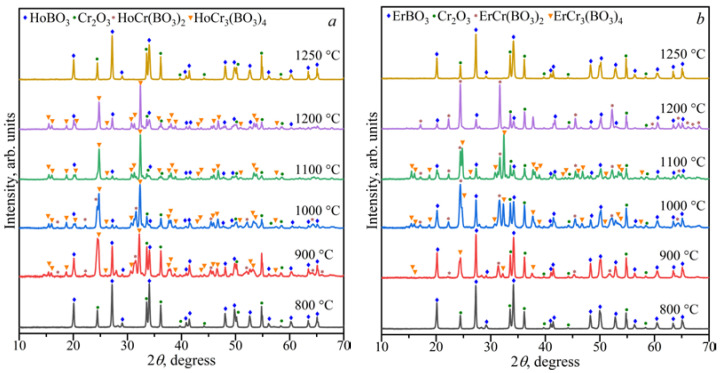
PXRD patterns of the Ho_2_O_3_:3Cr_2_O_3_:4B_2_O_3_ (**a**) and Er_2_O_3_:3Cr_2_O_3_:4B_2_O_3_ (**b**) compositions in the temperature range of 800–1250 °C.

**Figure 7:**
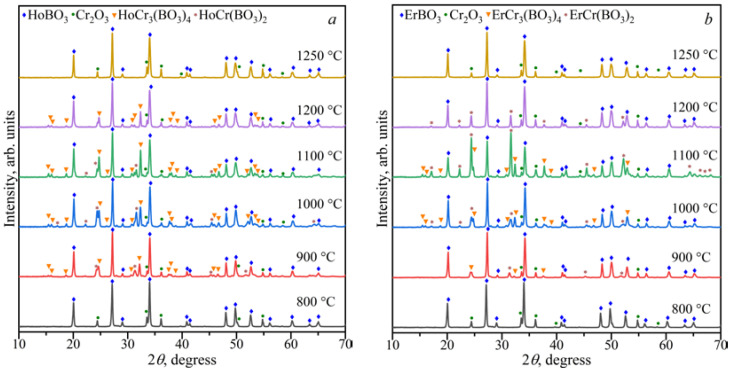
PXRD patterns of the Ho_2_O_3_:Cr_2_O_3_:2B_2_O_3_ (**a**) and Er_2_O_3_:Cr_2_O_3_:2B_2_O_3_ (**b**) compositions in the temperature range of 800–1250 °C.

The study of the stability of the borates *Ln*Cr_3_(BO_3_)_4_ and *Ln*Cr(BO_3_)_2_ in the range of 800–1250 °C showed that *Ln*BO_3_ and Cr_2_O_3_ were formed above 1200 °C (Figure 6 and Figure 7). The HoCr_3_(BO_4_)_3_, HoBO_3_, and Cr_2_O_3_ phases were stable at 1200 °C for the Ho_2_O_3_:3Cr_2_O_3_:4B_2_O_3_ and Ho_2_O_3_:Cr_2_O_3_:2B_2_O_3_ compositions. The ErCr(BO_3_)_2_, ErBO_3_, and Cr_2_O_3_ phases were stable at 1200 °C for the Er_2_O_3_:3Cr_2_O_3_:4B_2_O_3_ and Er_2_O_3_:Cr_2_O_3_:2B_2_O_3_ compositions. The *Ln*Cr_3_(BO_3_)_4_, *Ln*Cr(BO_3_)_2_, *Ln*BO_3_, and Cr_2_O_3_ phases coexisted from 900 to 1100 °C for the *Ln*_2_O_3_:3Cr_2_O_3_:4B_2_O_3_ (*Ln* = Ho, Er) and *Ln*_2_O_3_:Cr_2_O_3_:2B_2_O_3_ (*Ln* = Ho, Er) compositions. The *Ln*BO_3_ and Cr_2_O_3_ compounds were observed below 900 °C.

Thus, the following reactions occurred:

*Ln*_2_O_3_ + B_2_O_3_ → 2*Ln*BO_3_ (800–1250 °C) (7)

*Ln*_2_O_3_ + Cr_2_O_3_ + 2B_2_O_3_ → 2*Ln*Cr(BO_3_)_2_ (900–1200 °C for *Ln* = Er and 900–1100 °C for *Ln* = Ho) (8)

*Ln*_2_O_3_ + 3Cr_2_O_3_ + 4B_2_O_3_ → 2*Ln*Cr_3_(BO_3_)_4_ (900–1200 °C for *Ln* = Ho and 900–1100 °C for *Ln* = Er) (9)

#### 3.1.4. The *Ln*_2_O_3_–Cr_2_O_3_–B_2_O_3_ (*Ln* = Tm–Lu) Ternary Systems

**Figure 8:**
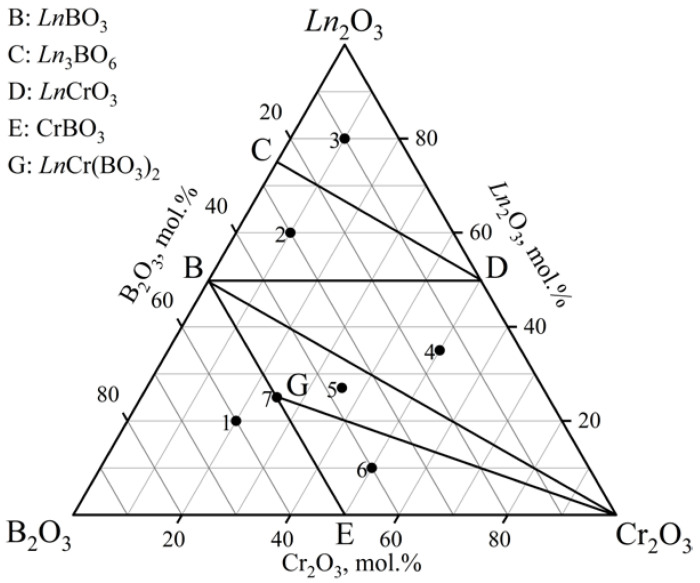
Phase relations in the *Ln*_2_O_3_–Cr_2_O_3_–B_2_O_3_ (*Ln* = Tm–Lu) system at 1000 °C.

The phase relations in the *Ln*_2_O_3_–Cr_2_O_3_–B_2_O_3_ (*Ln* = Tm–Lu) ternary systems were defined at 1000 °C (Table 4, Figure 8) based on the phase compositions for 21 samples (seven samples per system). There were six subsidiary subsystems. The double borates (TmCr(BO_3_)_2_ [PDF-2 #01-082-9335], YbCr(BO_3_)_2_ [PDF-2 #01-082-9336], and LuCr(BO_3_)_2_ [PDF-2 #01-082-9337]) were found. The orthoborates TmBO_3_ [PDF-2 #00-013-0482] and YbBO_3_ [PDF-2 #01-076-7335] were isostructural to the vaterite modification, and LuBO_3_ crystallized in the vaterite [PDF-2 #01-085-7631] and calcite [PDF-2 #01-085-7534] modifications. The Tm_2_O_3_, Yb_2_O_3_, and Lu_2_O_3_ phases were determined by using the powder data from the PDF-2 #00-041-1090, PDF-2 #00-041-1106, PDF-2 #00-012-0728 cards, respectively. The oxyborate Tm_3_BO_6_ was determined from the PXRD patterns of the isostructural compound Y_3_BO_6_, and *Ln*_3_BO_6_ (*Ln* = Yb, Lu) was established through similarity with the PXRD patterns from the literature [22].

Leonyuk et al. [39] reported the formation of YbCr_3_(BO_3_)_4_. Nevertheless, the existence of this phase is in doubt. In studies on the synthesis and characterization of *Ln*Cr_3_(BO_3_)_4_ compounds by using vibrational spectroscopy, the authors of [44,45,46] reported huntite-type borates with *Ln* = La, Pr, Nd, Sm–Er, which corresponded with our data.

**Figure 9:**
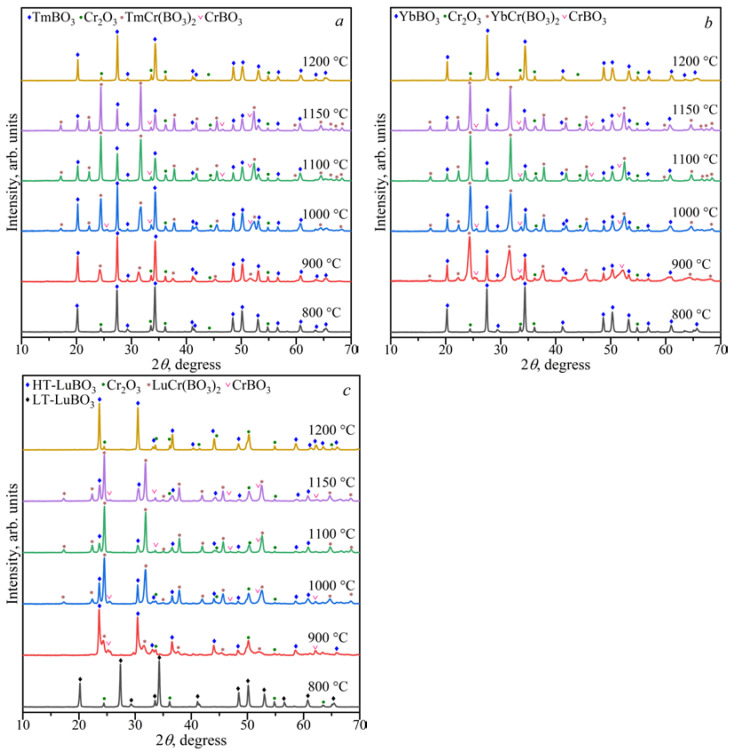
PXRD patterns of solids phases of the Tm_2_O_3_:Cr_2_O_3_:2B_2_O_3_ (**a**), Yb_2_O_3_:Cr_2_O_3_:2B_2_O_3_ (**b**), and Lu_2_O_3_:Cr_2_O_3_:2B_2_O_3_ (**c**) compositions in the range of 800–1200 °C.

The study of the stability *Ln*Cr(BO_3_)_2_ borates in the range of 800–1200 °C showed that above 1150 °C, the *Ln*BO_3_ and Cr_2_O_3_ phases formed (Figure 9). At temperatures from 900 to 1150 °C, *Ln*Cr(BO_3_)_2_ borates coexisted with *Ln*BO_3_, CrBO_3_, and Cr_2_O_3_. The *Ln*BO_3_ and Cr_2_O_3_ compounds were observed below 900 °C.

Therefore, the following reactions occurred:

*Ln*_2_O_3_ + B_2_O_3_ → 2*Ln*BO_3_ (800–1200 °C) (10)

Cr_2_O_3_ + B_2_O_3_ → 2CrBO_3_ (900–1150 °C) (11)

*Ln*_2_O_3_ + Cr_2_O_3_ + 2B_2_O_3_ → 2*Ln*Cr(BO_3_)_2_ (900–1150 °C) (12)

In the Lu_2_O_3_–Cr_2_O_3_–B_2_O_3_ system, at 800 °C, the borate LuBO_3_ was obtained only in a vaterite-type structure (LT modification of LuBO_3_); in the temperature range of 900–1000 °C, there were vaterite- and calcite-type structures, and at higher temperatures, they crystallized only in a calcite-type structure (HT modification of LuBO_3_). The obtained materials were consistent with the data obtained by the authors of [15]. It should be noted that the borate LuBO_3_ with only the calcite structure in the isothermal section at 1000 °C was observed for regions with a high content of boron oxide (regions 1 and 5 in Figure 8). The coexistence of calcite and vaterite modifications of the borate LuBO_3_ was observed in other regions.

### 3.2. Powder X-ray Diffraction

**Figure 10:**
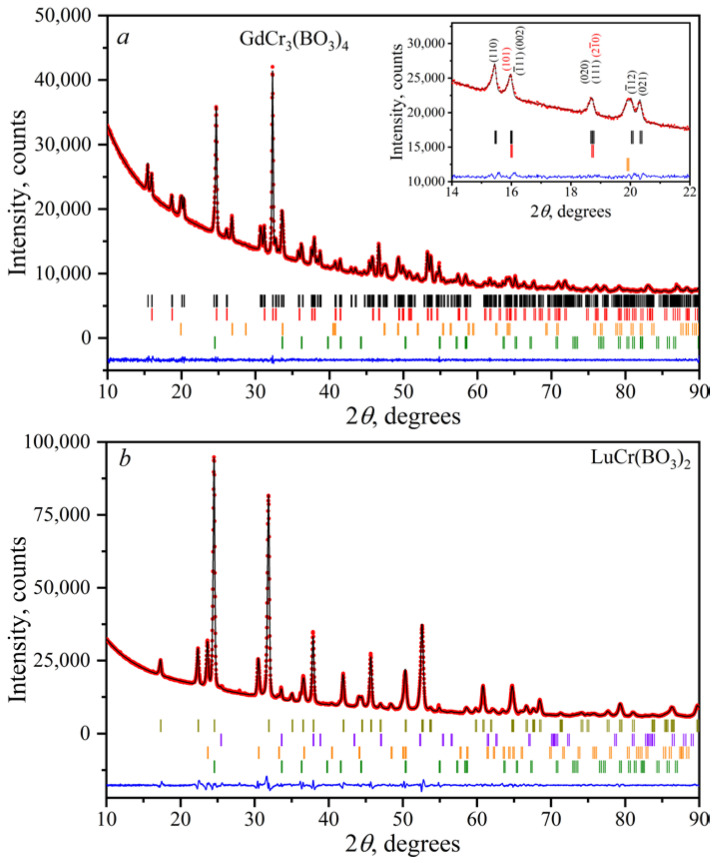
Final convergence of the Le Bail refinements for *α*-, *β*-GdCr_3_(BO_3_)_4_ (**a**) and LuCr(BO_3_)_2_ (**b**). The calculated and observed diffraction profiles are shown with a black solid line and red circles, respectively. The bottom blue trace is a plot of the difference between the calculated and observed intensities. Black, red, orange, green, dark yellow, and violet vertical marks denote Bragg reflections due to the main and impurity *β*-GdCr_3_(BO_3_)_4_, *α*-GdCr_3_(BO_3_)_4_, *Ln*BO_3_ (*Ln* = Gd, Lu), Cr_2_O_3_, LuCr(BO_3_)_2_, and CrBO_3_ phases, respectively.

The Le Bail fit was used to confirm the structural similarity of the borates within the families of *Ln*Cr_3_(BO_3_)_4_ and *Ln*Cr(BO_3_)_2_. All huntite-type *Ln*Cr_3_(BO_3_)_4_ borates turned out to be polytypes and contained a significant number of rhombohedral (sp. gr. *R*32, *α*-*Ln*Cr_3_(BO_3_)_4_) and monoclinic (sp. gr. *C*2/*c*, *β*-*Ln*Cr_3_(BO_3_)_4_) modifications. The refined lattice parameters are presented in Table 5 and Table 6 for *α*-, *β*-*Ln*Cr_3_(BO_3_)_4_ and *Ln*Cr(BO_3_)_2_, respectively. As an example, the convergences of the Le Bail fittings for *α*-, *β*-GdCr_3_(BO_3_)_4_ and LuCr(BO_3_)_2_ are shown in Figure 10. After the refinement, there was a good agreement between the calculated and experimental diffraction patterns with low-reliability factors. Peaks of impurity phases of *Ln*BO_3_, CrBO_3_, and Cr_2_O_3_ existed in the diffraction patterns. The decrease in the values of *a*, *b*, and *c* for both groups of compounds was due to a decrease in the ionic radii from Gd^3+^ to Er^3+^ [55]. The small deviation of the lattice parameters of *Ln*Cr(BO_3_)_2_ (Table 6) from those presented by Doi at el. [50] can be attributed to the disorder of *Ln*^3+^ and Cr^3+^ ions in the crystal structure.

**Figure 11:**
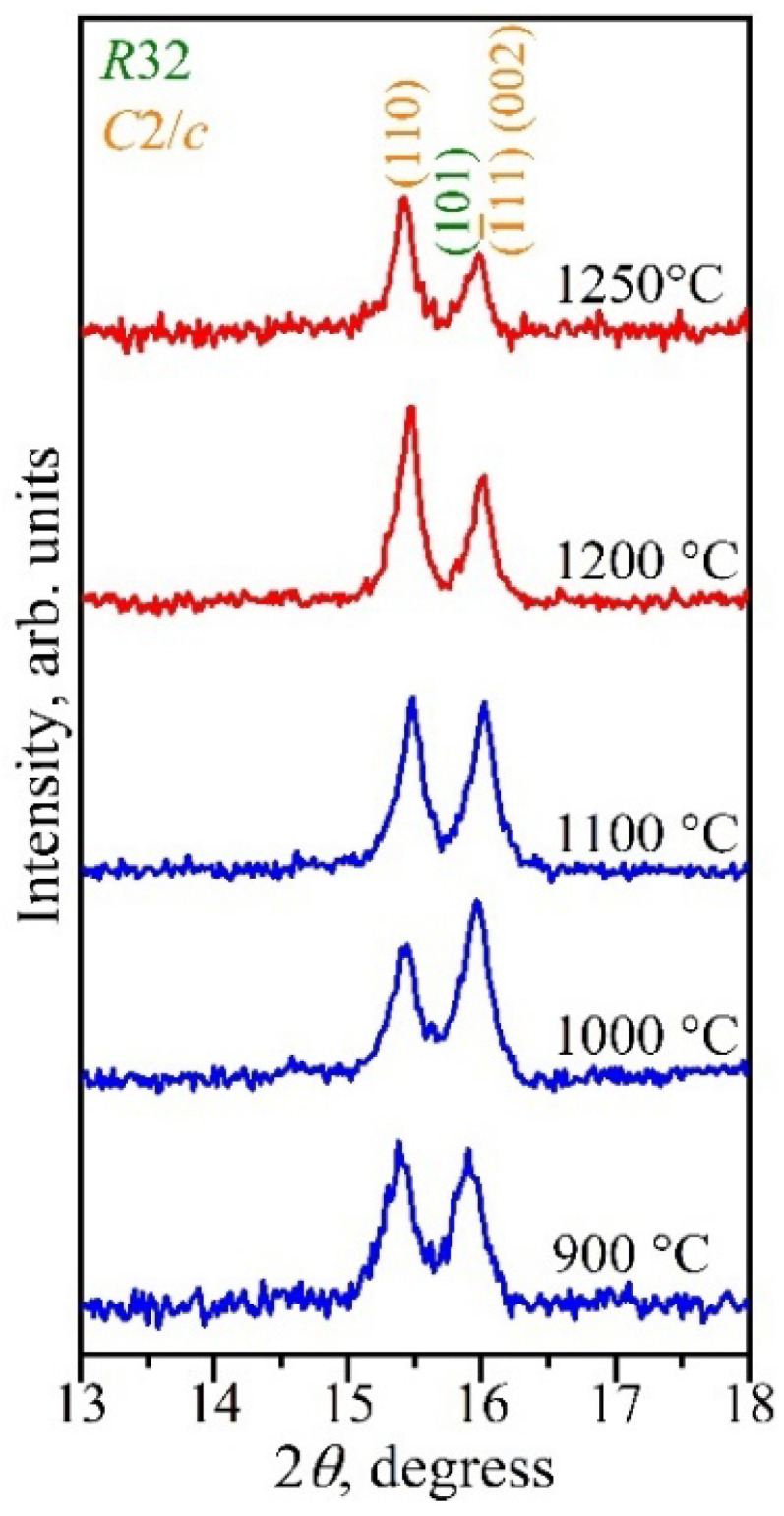
Bragg reflections $\left(110\right)$, $\left(101\right)$, $\left(\bar{1}11\right)$, and $\left(002\right)$ of α-DyCr_3_(BO_3_)_4_ and β-DyCr_3_(BO_3_)_4_ borates synthesized at different temperatures.

It is noted that with an increase in the synthesis temperature, the amount of the monoclinic *β*-*Ln*Cr_3_(BO_3_)_4_ modification increased, with a simultaneous decrease in the rhombohedral *α*-*Ln*Cr_3_(BO_3_)_4_ one. This is shown in Figure 11 by using DyCr_3_(BO_3_)_4_ as an example. Thus, this confirms the high-temperature nature of the monoclinic modification.

Mukherjee et al. [11] reported that they obtained a mixture of *α*-Dy(BO_2_)_3_ and DyBO_3_. However, they did not give structural parameters for the metaborate *α*-Dy(BO_2_)_3_. The metaborate *α*-Dy(BO_2_)_3_ was also refined with the Le Bail method. The lattice parameters were *a* = 6.1982 Å, *b* = 8.0213 Å, *c* = 7.8021 Å, *β* = 93.249°, and *V* = 386.27 Å^3^. *R*_wp_ = 0.77 and *R*_p_ = 1.03 became the final values of the *R*-factors. The additional phases were DyBO_3_ and Cr_2_O_3_. The obtained lattice parameters correlated with the trend towards a decrease in the lattice parameters of the *α*-*Ln*(BO_2_)_3_ (*Ln* = La–Nd, Sm–Tb) metaborates with the decrease in the ionic radius of the rare-earth ions [11].

### 3.3. Thermal Analysis

**Figure 12:**
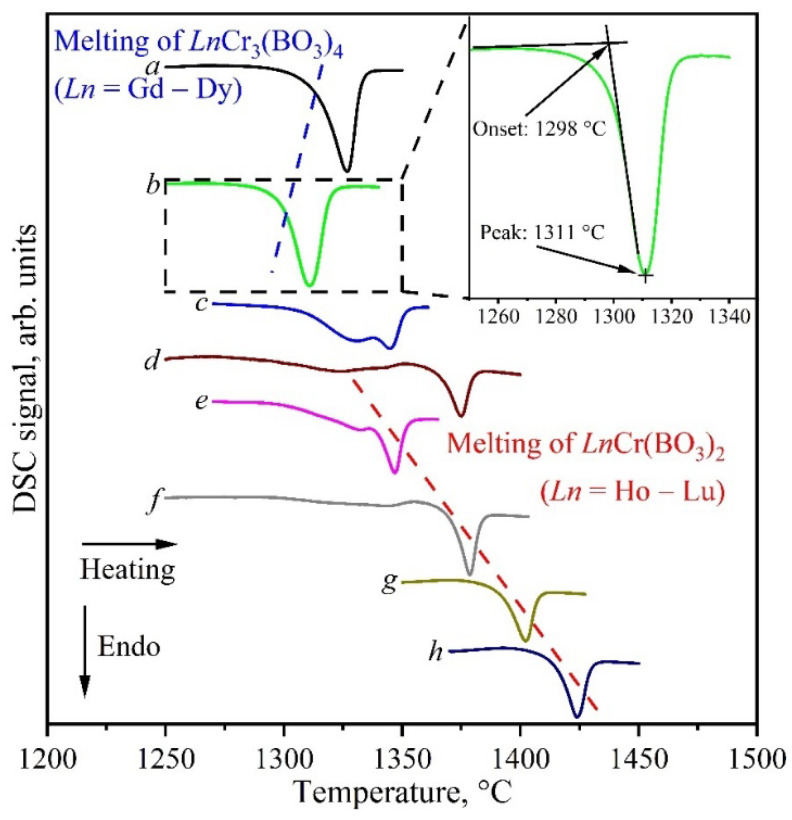
Fragments of the DSC curves of the *Ln*_2_O_3_:3Cr_2_O_3_:4B_2_O_3_ (*Ln* = Gd (*a*), Dy (*b*), Ho (*c*), Er (*d*)) and *Ln*_2_O_3_:Cr_2_O_3_:2B_2_O_3_ (*Ln* = Ho (*e*), Er (*f*), Tm (*g*), Yb (*h*)) compositions showing the melting points of the *Ln*Cr_3_(BO_3_)_4_ and *Ln*Cr(BO_3_)_2_ borates (the inset shows the notation used at the melting peak).

A thermal analysis was carried out to determine the melting points of rare-earth chromium borates. Figure 12 shows fragments of the DSC curves for the *Ln*_2_O_3_:3Cr_2_O_3_:4B_2_O_3_ (*Ln* = Gd–Er) and *Ln*_2_O_3_:Cr_2_O_3_:2B_2_O_3_ (*Ln* = Ho–Lu) compositions in the range of the melting points of the *Ln*Cr_3_(BO_3_)_4_ and *Ln*Cr(BO_3_)_2_ borates. The DSC curves had one or two endopeaks. One peak was observed for the *Ln*_2_O_3_:3Cr_2_O_3_:4B_2_O_3_ (*Ln* = Gd–Dy) and *Ln*_2_O_3_:Cr_2_O_3_:2B_2_O_3_ (*Ln* = Tm–Lu) compositions, and two were observed for the *Ln*_2_O_3_:3Cr_2_O_3_:4B_2_O_3_ (*Ln* = Ho, Er) and *Ln*_2_O_3_:Cr_2_O_3_:2B_2_O_3_ (*Ln* = Ho, Er) compositions. In the first case, one peak was explained by the melting of *Ln*Cr_3_(BO_3_)_4_ or *Ln*Cr(BO_3_)_2_ (the melting points are presented in Table 7). In the second case, the picture was more complicated. The coexistence of two borates, *Ln*Cr_3_(BO_3_)_4_ and *Ln*Cr(BO_3_)_2_, was found for the *Ln*_2_O_3_:3Cr_2_O_3_:4B_2_O_3_ (*Ln* = Ho, Er) and *Ln*_2_O_3_:Cr_2_O_3_:2B_2_O_3_ (*Ln* = Ho, Er) compositions (Section 3.1). Thus, the two peaks on the DSC curve were explained by the alternate melting of these two compounds. Table 7 shows that the melting point of the *Ln*Cr_3_(BO_3_)_4_ (*Ln* = Gd–Dy) borates gradually decreased as the atomic number of the rare-earth element in these compounds increased. At the same time, the melting point of the *Ln*Cr(BO_3_)_2_ (*Ln* = Tm–Lu) borates increased. Based on this, we attributed the low-temperature peak in the DSC curves to *Ln*Cr_3_(BO_3_)_4_ (*Ln* = Ho, Er), and we attributed the high-temperature peak to *Ln*Cr(BO_3_)_2_ (*Ln* = Ho, Er). In this case, we were guided by the peak value of the melting temperature, since, in our opinion, it reflected it better (there was a very wide peak for the ErCr_3_(BO_3_)_4_ borate, which was possibly due to the polycrystalline nature of the samples).

According to DSC and PXRD data (Section 3.1), the thermally induced processes can be illustrated with the following reactions:

2*Ln*Cr_3_(BO_3_)_4_ → 2*Ln*BO_3_ + 3Cr_2_O_3_ + 3B_2_O_3_ (*Ln* = Gd–Dy) (1310–1330 °C) (13)

4*Ln*Cr_3_(BO_3_)_4_ → 2*Ln*Cr(BO_3_)_2_ + 2*Ln*BO_3_ + 5Cr_2_O_3_ + 5B_2_O_3_ (*Ln* = Ho–Er) (1320–1330 °C) (14)

2*Ln*Cr(BO_3_)_2_ → 2*Ln*BO_3_ + Cr_2_O_3_ + B_2_O_3_ (*Ln* = Ho–Lu) (1330–1450 °C) (15)

## 4. Conclusions

The *Ln*_2_O_3_–Cr_2_O_3_–B_2_O_3_ (*Ln* = Gd–Lu) ternary oxide systems were studied with PXRD. In these systems, it was found that the *Ln*Cr_3_(BO_3_)_4_ borates were stable up to *Ln* = Er, after which *Ln*Cr(BO_3_)_2_ borates crystallized. It was established that *Ln*Cr_3_(BO_3_)_4_ borates crystallized in rhombohedral (*α*-*Ln*Cr_3_(BO_3_)_4_) and monoclinic (*β*-*Ln*Cr_3_(BO_3_)_4_) polytype modifications. The latter prevailed at temperatures above 1100 °C. The melting points of *Ln*Cr_3_(BO_3_)_4_ (*Ln* = Gd–Er) were slightly higher than those for *Ln*Al_3_(BO_3_)_4_ (*Ln* = Gd–Er). They increased with a decrease in the ionic radius of rare-earth ions for *Ln*Cr(BO_3_)_2_ (*Ln* = Ho–Lu) borates, while for *Ln*Cr_3_(BO_3_)_4_ (*Ln* = Gd–Er) borates, they decreased to *Ln* = Dy and then increased.

## Figures and Tables

**Table 1:** Phase compositions of specimens in the *Ln*_2_O_3_–Cr_2_O_3_–B_2_O_3_ (*Ln* = Gd, Tb) systems as indicated by PXRD.

Samples	*Ln*_2_O_3_, mol.%	Cr_2_O_3_, mol.%	B_2_O_3_, mol.%	Phase Composition
1	10	30	60	*Ln*Cr_3_(BO_3_)_4_ + CrBO_3_ + *Ln*(BO_2_)_3_ ^a^ + *Ln*BO_3_ ^b,c^
2	30	10	60	*Ln*Cr_3_(BO_3_)_4_ + *Ln*(BO_2_)_3_ ^a,d^ + *Ln*BO_3_
3	60	10	30	*Ln*BO_3_ + *Ln*CrO_3_ + *Ln*_3_BO_6_
4	80	10	10	*Ln*_2_O_3_ + *Ln*CrO_3_ + *Ln*_3_BO_6_
5	35	50	15	*Ln*BO_3_ + *Ln*CrO_3_ + Cr_2_O_3_
6	20	45	35	*Ln*Cr_3_(BO_3_)_4_ + Cr_2_O_3_ + *Ln*BO_3_
7	4	53	43	*Ln*Cr_3_(BO_3_)_4_ + CrBO_3_ + Cr_2_O_3_
8	12.5	37.5	50	*Ln*Cr_3_(BO_3_)_4_ + *Ln*BO_3_ + Cr_2_O_3_ + *Ln*(BO_2_)_3_ ^d,e^

^a^ At 900 and 1000 °C for *Ln* = Gd. ^b^ At 1100 °C for *Ln* = Gd. ^c^ At 900, 1000, 1100 °C for *Ln* = Tb. ^d^ At 900 °C for *Ln* = Tb. ^e^ At 900 °C for *Ln* = Tb.

**Table 2:** Phase compositions of specimens in the Dy_2_O_3_–Cr_2_O_3_–B_2_O_3_ system as indicated by PXRD.

Samples	Dy_2_O_3_, mol.%	Cr_2_O_3_, mol.%	B_2_O_3_, mol.%	Phase Composition
1	10	30	60	DyCr_3_(BO_3_)_4_ + CrBO_3_ + DyBO_3_
2	60	10	30	DyBO_3_ + DyCrO_3_ + Dy_3_BO_6_
3	80	10	10	Dy_2_O_3_ + DyCrO_3_ + Dy_3_BO_6_
4	35	50	15	DyBO_3_ + DyCrO_3_ + Cr_2_O_3_
5	20	45	35	DyCr_3_(BO_3_)_4_ + Cr_2_O_3_ + DyBO_3_
6	4	53	43	DyCr_3_(BO_3_)_4_ + CrBO_3_ + Cr_2_O_3_
7	12.5	37.5	50	DyCr_3_(BO_3_)_4_ + DyBO_3_ + Cr_2_O_3_

**Table 3:** Phase compositions of specimens in the *Ln*_2_O_3_–Cr_2_O_3_–B_2_O_3_ (*Ln* = Ho, Er) systems as indicated by PXRD.

Samples	*Ln*_2_O_3_, mol.%	Cr_2_O_3_, mol.%	B_2_O_3_, mol.%	Phase Composition
1	15	25	60	*Ln*Cr_3_(BO_3_)_4_ + *Ln*Cr(BO_3_)_2_ + CrBO_3_ + *Ln*BO_3_
2	60	10	30	*Ln*BO_3_ + *Ln*CrO_3_ + *Ln*_3_BO_6_
3	80	10	10	*Ln*_2_O_3_ + *Ln*CrO_3_ + *Ln*_3_BO_6_
4	35	50	15	*Ln*BO_3_ + *Ln*CrO_3_ + Cr_2_O_3_
5	27	36	37	*Ln*Cr(BO_3_)_2_ + Cr_2_O_3_ + *Ln*BO_3_
6	18	36	46	*Ln*Cr_3_(BO_3_)_4_ + *Ln*Cr(BO_3_)_2_ + Cr_2_O_3_
7	4	53	43	*Ln*Cr_3_(BO_3_)_4_ + CrBO_3_ + Cr_2_O_3_
8	12.5	37.5	50	*Ln*Cr_3_(BO_3_)_4_ + *Ln*BO_3_+ Cr_2_O_3_
9	25	25	50	*Ln*Cr_3_(BO_3_)_4_ + *Ln*Cr(BO_3_)_2_ + Cr_2_O_3_ + *Ln*BO_3_

**Table 4:** Phase compositions of specimens in the *Ln*_2_O_3_–Cr_2_O_3_–B_2_O_3_ (*Ln* = Tm–Lu) systems as indicated by PXRD.

Samples	*Ln*_2_O_3_, mol.%	Cr_2_O_3_, mol.%	B_2_O_3_, mol.%	Phase Composition
1	20	20	60	*Ln*Cr(BO_2_)_3_ + CrBO_3_ + *Ln*BO_3_ ^a^
2	60	10	30	*Ln*BO_3_ ^b^ + *Ln*CrO_3_ + *Ln*_3_BO_6_
3	80	10	10	*Ln*_2_O_3_ + *Ln*CrO_3_ + *Ln*_3_BO_6_
4	35	50	15	*Ln*BO_3_ ^b^ + *Ln*CrO_3_ + Cr_2_O_3_
5	27	36	37	*Ln*Cr(BO_3_)_2_ + Cr_2_O_3_ + *Ln*BO_3_ ^a^
6	10	50	40	*Ln*Cr(BO_3_)_2_ + CrBO_3_ + Cr_2_O_3_
7	25	25	50	*Ln*Cr(BO_3_)_2_ + *Ln*BO_3_ ^a^ + CrBO_3_ + Cr_2_O_3_

^a^ Calcite modification for *Ln* = Lu. ^b^ Calcite modification for *Ln* = Lu.

**Table 5:** Lattice parameters of *α*-, *β*-*Ln*Cr_3_(BO_3_)_4_ (*Ln* = Gd–Er) borates (sp. gr. *R*32 and sp. gr. *C*2*/c*).

Refined Parameters	*β*-GdCr_3_(BO_3_)_4_	*β*-TbCr_3_(BO_3_)_4_	*β*-DyCr_3_(BO_3_)_4_	*β*-HoCr_3_(BO_3_)_4_	*β*-ErCr_3_(BO_3_)_4_
*a* (Å)	7.3939	7.3912	7.3828	7.3741	7.3712
*b* (Å)	9.4873	9.4846	9.4763	9.4728	9.4647
*c* (Å)	11.4031	11.3934	11.3782	11.3773	11.3731
*β* (°)	103.853	103.861	103.847	103.843	103.852
*V* (Å^3^)	776.63	775.45	772.90	771.66	770.38
	*α*-GdCr_3_(BO_3_)_4_	*α*-TbCr_3_(BO_3_)_4_	*α*-DyCr_3_(BO_3_)_4_	*α*-HoCr_3_(BO_3_)_4_	*α*-ErCr_3_(BO_3_)_4_
*a* (Å)	9.4700	9.4693	9.4657	9.4650	9.4637
*c* (Å)	7.4976	7.4959	7.4801	7.4747	7.4684
*V* (Å^3^)	582.31	582.09	580.42	579.92	579.26
*R*_wp_ (%)	1.01	1.13	1.13	1.33	1.51
*R*_p_ (%)	0.76	0.85	0.83	0.95	1.04
T_synthesis_ (°C)	1200	1200	1200	1200	1100

**Table 6:** Lattice parameters of LnCr(BO_3_)_2_ (Ln = Ho–Lu) borates (sp. gr. $R\bar{3}$ ).

Refined Parameters	HoCr(BO_3_)_2_	ErCr(BO_3_)_2_	TmCr(BO_3_)_2_	YbCr(BO_3_)_2_	LuCr(BO_3_)_2_
*a* (Å)	4.7664	4.7591	4.7528	4.7471	4.7442
*c* (Å)	15.5415	15.490	15.4835	15.4061	15.3471
*V* (Å^3^)	305.77	303.84	302.90	300.66	299.14
*R*_wp_ (%)	1.75	3.19	3.15	2.83	2.36
*R*_p_ (%)	1.20	2.00	2.04	1.79	1.58
T_synthesis_ (°C)	1000	1200	1100	1100	1100

**Table 7:** Melting points of t*he Ln*Cr_3_(BO_3_)_4_ (*Ln* = Gd–Er) and *Ln*Cr(BO_3_)_2_ (*Ln* = Ho–Lu) borates.

Compound	Peak (°C)	Onset (°C)
GdCr_3_(BO_3_)_4_	1327	1312
TbCr_3_(BO_3_)_4_	1321	1303
DyCr_3_(BO_3_)_4_	1311	1298
HoCr_3_(BO_3_)_4_	1331	1302
ErCr_3_(BO_3_)_4_	1343	1263
HoCr(BO_3_)_2_	1347	1333
ErCr(BO_3_)_2_	1379	1366
TmCr(BO_3_)_2_	1402	1391
YbCr(BO_3_)_2_	1424	1413
LuCr(BO_3_)_2_	>1500	1448

## Data Availability

Data can be obtained from the corresponding author upon reasonable request.
